# Five Novel Mutations in LOXHD1 Gene Were Identified to Cause Autosomal Recessive Nonsyndromic Hearing Loss in Four Chinese Families

**DOI:** 10.1155/2020/1685974

**Published:** 2020-02-18

**Authors:** Xiaohui Bai, Chi Zhang, Fengguo Zhang, Yun Xiao, Yu Jin, Haibo Wang, Lei Xu

**Affiliations:** ^1^Otologic Center, Shandong Provincial ENT Hospital Affiliated to Shandong University, Jinan, China; ^2^Department of Clinical Laboratory, Shandong Provincial Hospital Affiliated to Shandong University, Jinan, China

## Abstract

Hearing loss is one of the most common sensory disorders in newborns and is mostly caused by genetic factors. Autosomal recessive nonsyndromic hearing loss (ARNSHL) is usually characterized as a severe-to-profound congenital sensorineural hearing loss and later can cause various degrees of defect in the language and intelligent development of newborns. The mutations in *LOXHD1* gene have been shown to cause DFNB77, a type of ARNSHL. To date, there are limited reports about the association between *LOXHD1* gene and ARNSHL. In this study, we reported six patients from four Chinese families suffering from severe-to-profound nonsyndromic hearing loss. We performed targeted next generation sequencing in the six affected members and identified five novel pathogenic mutations in *LOXHD1* including c.277G>A (p.D93N), c.611-2A>T, c.1255+3A>G, c.2329C>T (p.Q777^*∗*^), and c.5888delG (p.G1963Afs^*∗*^136). These mutations were confirmed to be cosegregated with the hearing impairment in the families by Sanger sequencing and were inherited in an autosomal recessive pattern. All of the five mutations were absent in 200 control subjects. There were no symptoms of Fuchs corneal dystrophy in the probands and their blood-related relatives. We concluded that these five novel mutations could be involved in the underlying mechanism resulting in the hearing loss, and this discovery expands the genotypic spectrum of *LOXHD1* mutations.

## 1. Introduction

Hearing loss is one of the most common sensory diseases, affecting the life quality of 466 million individuals in the world (http://www.who.int/pbd/deafness/estimates/en/). Totally about 278 million people suffer from hearing impairments, and the incidence of hearing loss is 1 to 3 per 1,000 for newborns [1]. Previous studies suggested that deafness is associated with genetic factors accounting for at least 60% of the cases of deafness [2].

According to the symptoms, hereditary hearing loss can be divided into two categories, syndromic (30%) and nonsyndromic (70%) [3]. However, based on the inheritance pattern, hereditary hearing loss is classified into four types, including autosomal recessive, autosomal dominant, mitochondrial, and X-linked inheritance. Among them, ARNSHL is the most common disease and accounts for approximately 80% of cases in NSHL. ARNSHL is usually characterized as congenital, nonsyndromic, severe-to-profound, and nonprogressive sensorineural hearing loss. The most well-known pathogenic gene related to ARNSHL is GJB2 [4]. So far, over 70 genes have been identified to associate with ARNSHL (http://hereditaryhearingloss.org).

Lipoxygenase homology domains 1 (*LOXHD1*) gene mapped on chromosome 18q12-21 encodes a protein that localizes along the plasma membrane of stereocilia in the hair cells [5], and mutations in *LOXHD1* gene have been shown to cause DFNB77, a type of ARNSHL. The protein encoded by *LOXHD1* contains 2,211 amino acids and 15 polycystin-1/lipoxygenase/alpha-toxin (PLAT) domains which are believed to be involved in targeting its protein to the plasma membrane. It played an important role in maintaining normal hair cell function in the cochlea [5].

Studies on the association between *LOXHD1* mutations and hereditary hearing loss are limited. At present, less than 80 different pathogenic variants were reported with seven of these identified in Chinese population [6–8]. Therefore, studies are needed to reveal the potential genotype-phenotype correlations between *LOXHD1* mutations and ARNSHL.

LOXHD1 mutation-related DFNB77 is called nonsyndromic hearing loss and contains no other symptoms. Moreover, studies suggested that a single heterozygous mutation of LOXHD1 was also associated with another hereditary disease, Fuchs corneal dystrophy (FCD) [9]. Other studies demonstrated conflicting conclusions that there was no association between LOXHD1 mutations and FCD [9, 10].

In this study, we identified five novel mutations in *LOXHD1* which were predicted to be the pathogenic variants from four Chinese families suffering from ARNSHL.

## 2. Materials and Methods

### 2.1. Research Subjects and Clinical Evaluation

**Figure 1:**
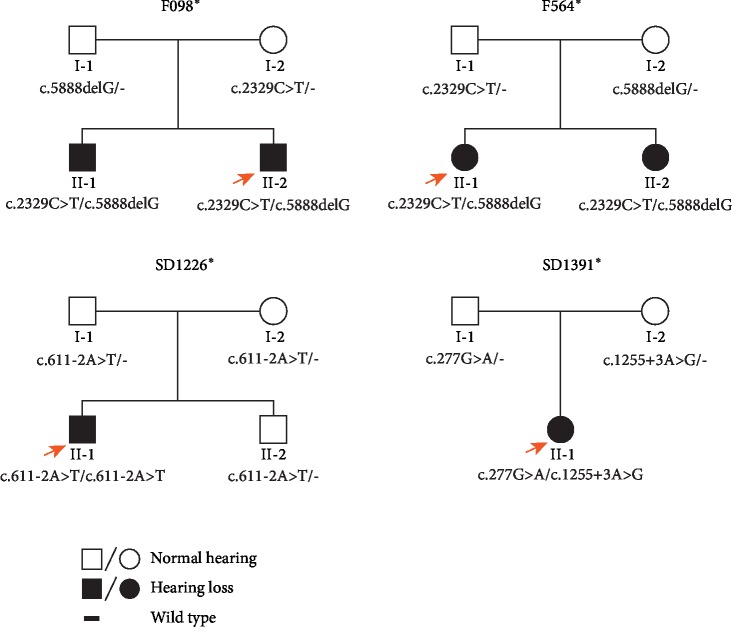
Pedigrees of the hearing loss families and identified pathogenic variants. Black squares represent members with hearing loss. Genotypes are marked below each member. Arrow shows the proband. Asterisks indicate the families with LOXHD1 mutations identified in the present study.

Four Chinese families with autosomal recessive sensorineural hearing loss were recruited in the Otologic Center, Shandong Provincial ENT Hospital Affiliated to Shandong University (Figure 1). All the members in the four families received clinical examinations in our hospital.

All participants received ophthalmological and auditory tests, including pure tone audiometry (PTA), distortion product otoacoustic emission, auditory brainstem response, acoustic immittance, and tinnitus examination. The guideline of American Speech-Language-Hearing Association was used to determine the degrees of hearing loss [11]. For the members under the age of 3, the hearing was assessed through behavior observation audiometry. The degree of deafness was classified into 5 grades, normal hearing (PTA ≤ 25 dB·HL), mild (26 ≤ PTA ≤ 40 dB·HL), moderate (41 ≤ PTA ≤ 60 dB·HL), severe (61 ≤ PTA ≤ 80 dB·HL), and profound hearing loss (PTA ≥ 81 dB HL), based on PTA threshold applied to the ear with better hearing at 250, 500, 1000, 2000, 4000, and 8000 Hz [12].

Prior to the study, the informed consent forms were provided to and signed by all the family members. Our study was approved by the ethics committee of the Institutional Review Board of Shandong Provincial ENT Hospital Affiliated to Shandong University (XYK20140101).

### 2.2. Genetic Analysis

In order to discover the pathogenic variants in the four Chinese families, a DNA extraction kit was used to extract genomic DNA from the peripheral blood (Axygen, USA). Targeted next generation sequencing was used to screen mutations from 127 genes (Supplementary [Supplementary-material supplementary-material-1]) related to hereditary hearing loss in the genomic DNA of the probands. The sequencing library covered 127 genes related to nonsyndromic hearing loss. A standardized next generation sequencing platform was applied and data were analyzed by BGIv0.1.0. Also, BGIv0.1.0 needs a reference. BWA 0.6.2-r126 software was used to align the reads to the human reference genome UCSC hg19 Feb.2009. GATK was used for mutation detection with dbSNP (snp137) used as a reference. The novel pathogenic variations were investigated by the 1000 genome database (Phase I) (http://www.1000genomes.org) and HapMap database (combined data from Phases II and III). We referred to the American Medical Genetics and Genomics Guide to interpret data [13].

### 2.3. Mutation Confirmation by Sanger Sequencing

We performed Sanger sequencing to verify the mutations in subjects and 200 controls. PCR was employed to amplify the regions corresponding to these mutations (Table 1). LOXHD1 mRNA (RefSeq NM_144612.6) and corresponding protein sequence (NP_653213.6) were used as a reference to align the sequences with Lasergene-SeqMan software.

## 3. Results

### 3.1. Clinical Manifestations

The hearing loss of the four Chinese pedigrees from Shandong Province enrolled in this study (Figure 1) is an autosomal recessive disease. Most of the affected patients were given a diagnosis in their newborn screening and suffered hearing loss after birth. However, the patient SD1226^*∗*^ II-1 was first diagnosed as hearing loss at age 3.

**Figure 2:**
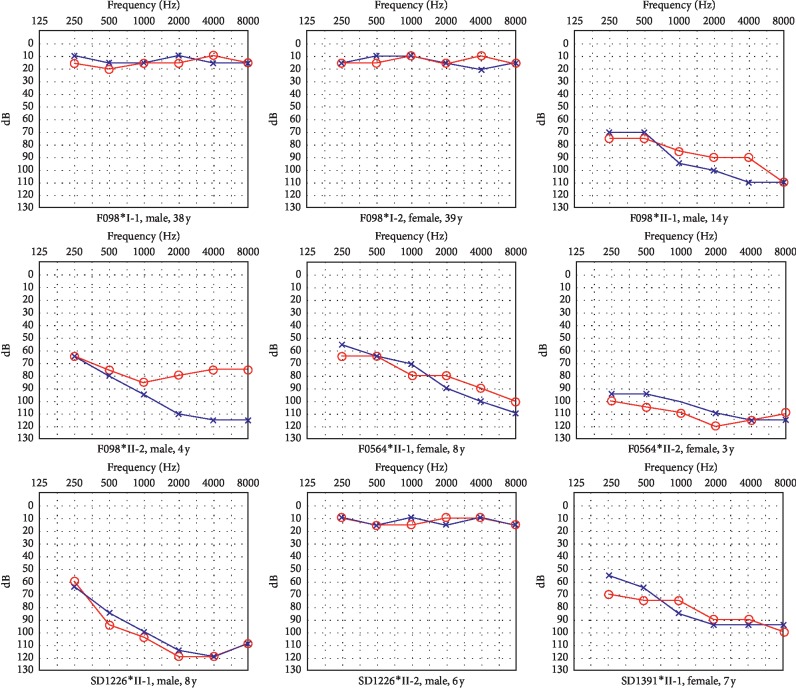
Audiograms of some members participating in this study in the four Chinese families. Blue crosses and red circles represent the air conduction hearing threshold levels of left and right ears, respectively. Asterisks indicate the families with LOXHD1 mutations identified in this study. Gender and age are shown below the audiogram of each individual.

All the hearing loss patients demonstrated bilaterally symmetrical, severe-to-profound sensorineural hearing impairment at all frequencies, but predominantly at middle to high frequencies based on the PTA test (Figure 2). Bilaterally symmetrical hearing loss was indicated as when the difference between the hearing thresholds of both ears was less than 10 dB at three or more frequencies or less than 15 dB at two or more frequencies. The degree of hearing loss did not increase with age according to their statements. Moreover, all the parents of the affected patients have normal hearing. Clinical features of some participants are given in Table 2.

None of the affected members declared that they underwent tinnitus and vertigo. After comprehensive physical and otologic examinations, all the other abnormalities and systematic disorders were excluded. All parents denied the use of any ototoxic medications and the occurrence of viral infection during pregnancy.

All the family members enrolled in this study denied the symptoms of eye pain, glare, or blurred vision. The probands and their blood-related relatives in F098^*∗*^ family showed no corneal abnormalities (Supplementary [Supplementary-material supplementary-material-1]).

### 3.2. Novel Mutations in LOXHD1 Gene Were Demonstrated to Cause ARNSHL

**Figure 3:**
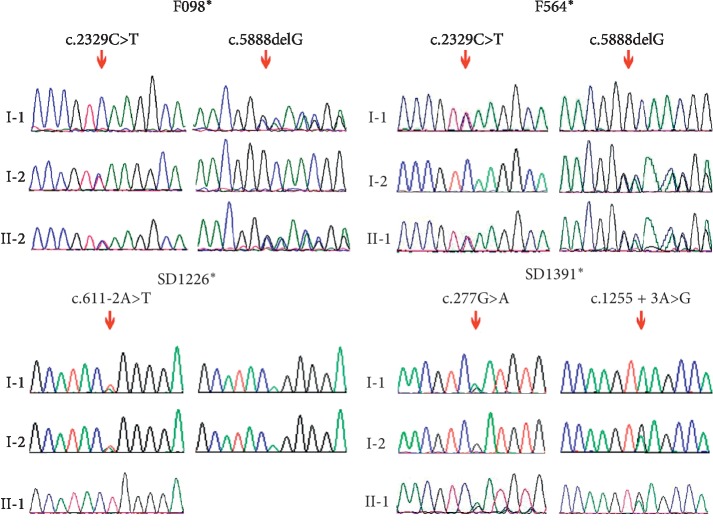
Sanger sequencing results of the probands in the four families. Red arrows point to the positions of the LOXHD1 mutations.

**Figure 4:**
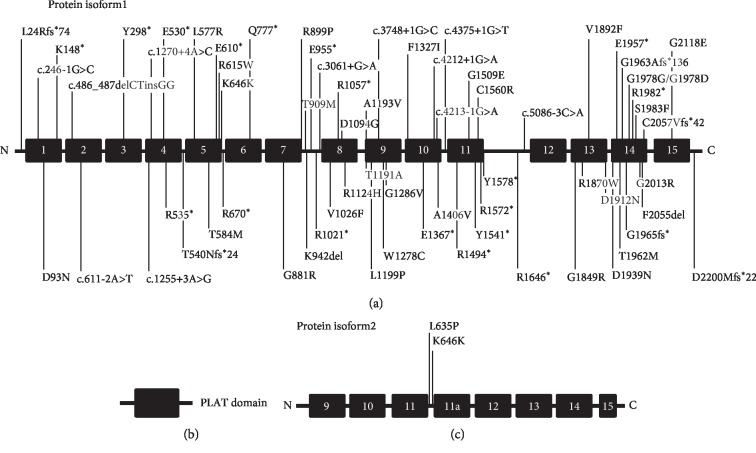
All identified pathogenic variants in LOXHD1 gene associated with DFNB77. (a) Isoform 1 represents LOXHD1 protein NP_653213.6. (b) Schematic representation of PLAT protein domain. (c) Isoform 2 represents LOXHD1 protein NP_001138944.1. Two variants (L635P and splice site variants K646K) only affect the shorter isoform 2. The blue label represents the previously reported mutations causing DFNB77 deafness, while the red label represents novel mutations in this work [14].

Five novel mutations (family F098^*∗*^, F564^*∗*^, SD1226^*∗*^, and SD1391^*∗*^) in *LOXHD1* gene were identified pathogenic variants based on predictive analysis using PolyPhen2, SIFT, and Mutation Taster. Sanger sequencing was used in all the subjects to verify variants in *LOXHD1*. The c.2329C>T (p.Q777X) and c.5888delG (p.G1963Afs^*∗*^136) mutations were both found in family F098^*∗*^ and family F564^*∗*^. The c.611-2A>T mutation was verified in family SD1226^*∗*^, while c.277G>A (p.D93N) and c.1255+3A>G were verified in family SD1391^*∗*^. Sequencing results are shown in Figures 2 and 3, and the schematic diagrams of protein structure are shown in Figure 4.

In general, according to ACMG guidelines [13], the mutations of c.611-2A>T, c.1255+3A>G, c.2329C>T (p.Q777^*∗*^), and c.5888delG (p.G1963Afs^*∗*^136) were classified as pathogenic; in addition, the mutation of c.277G>A (p.D93N) was classified as likely pathogenic. Moreover, all the mutations were newly identified and never reported previously (Figure 4). Those mutations were absent in all of 200 control subjects with the method of Sanger sequencing.

The mutation of c.2329C>T (p.Q777X) is a nonsense mutation, which leads to a stop codon in PLAT 6 domain. The variant c.277G>A (p.D93N) is also a missense mutation found in PLAT 1 domain. But the mutation of c.5888delG (p.G1963Afs^*∗*^136) is a frameshift mutation in PLAT 14 domain, resulting in a truncated protein of *LOXHD1*. In addition, the mutations of c.611-2A>T and c.1255+3A>G can cause defects in alternative gene splicing of PLAT 2 and PLAT 4 domain, respectively. Figure 4 shows all the previously reported mutations in *LOXHD1* that cause DFNB77-type deafness, as well as novel mutations identified in this study. These results showed that *LOXHD1* mutations are found throughout all PLAT domains.

## 4. Discussion

In our study, five novel mutations in *LOXHD1* gene were reported in four Chinese families with six members suffering from bilateral sensorineural hearing loss. Studies suggest that *LOXHD1* gene mutation can cause DFNB77-type deafness, which is characterized as a congenital or delayed hearing loss in an autosomal recessive inheritance pattern [6–8].

The *LOXHD1* gene encodes a 2211 amino-acid highly conserved protein and includes 15 PLAT domains [15]. At present, the function of LOXHD1 protein has not been clarified. The LOXHD1 protein is predominantly located along the membrane of hair cell stereocilia, indicating its role in maintaining normal hearing [5]. Grillet et al. described a recessive *LOXHD1* mutation in the samba mouse line, which was generated in an ethylnitrosourea (ENU) mutagenesis screen [5]. The homozygous samba mice showed impaired hearing but intact vestibular function. The expression of LOXHD1 protein was detected at both cochlear and vestibular hair cells. Although the development of hair cell stereocilia was unaffected, the function of hair cells was impaired.

In general, hearing loss caused by *LOXHD1* was a sensorineural hearing loss at mid-to-high frequencies with no vestibular symptoms [6–8]. They showed either stable or progressive hearing loss. The severity of hearing loss varied from mild to profound level. To date, the reports of LOXHD1 mutations related to ARNSHL are limited and no functional experiments have been done to confirm the pathogenesis of these mutations. As shown in Table 3, the reported mutations of *LOXHD1* were limited and these mutations are relatively rare in Chinese population.

Normally, ARNSHL is characterized as a congenital hearing loss. However, previous studies have shown that the onset age of LOXHD1-related hearing loss had a large span, ranging from newborn, prelingual, childhood to even adulthood. Eppsteiner et al. reported a patient with compound heterozygous *LOXHD1* mutations, whose hearing loss started at age 40 and gradually aggravated till 67 years old [27]. The progression of hearing loss was generally determined by self-report which might not be reliable. Minami et al. reported successive results of hearing examinations [29]. Two members in a Japanese family with compound heterozygous mutations in *LOXHD1* suffered from progressive hearing loss. Member III-2 was assessed by PTA at age of 7 y 4 m, 9 y 8 m, and 10 y 5 m, while the proband III-3 was assessed via conditional orientation response audiometry at age of 1 y 11 m, 2 y 5 m, 3 y 10 m, and 4 y 10 m. They were both diagnosed with slowly aggravated hearing loss. Further follow-up is needed to monitor the progression of hearing loss for these two patients [29].

FCD is characterized as bilateral corneal guttae, corneal edema, discomfort, and blurred vision caused by a reduced density of endothelial cells [31]. The onset of FCD is usually during the 4th decade of life and progresses slowly. In 2012, Riazuddin et al. [9] reported that the patients with FCD carried more mutations in *LOXHD1* than the controls. They concluded that *LOXHD1* mutations may be related to dominant late onset of FCD [9]. Tang et al. studied five genes related to FCD including LOXHD1 and revealed that only two mutations were found in the intron regions of LOXHD1 in a Chinese pedigree suffering from FCD [11].

Recently, Stehouwer et al. investigated 72 FCD patients and discovered that 45.8% of them had hearing disability, which was much higher than the control group [32]. However, in the four families we have studied, all the patients and the family members with normal hearing but carrying a single heterozygous mutation in the *LOXHD1* gene showed no corneal abnormalities. Other studies also reported no symptoms of FCD were observed in the probands and their blood-related relatives [7, 10, 33]. Although the relationship of *LOXHD1*-related hearing loss and FCD is still unclear, we suggest that ophthalmologic examinations should be performed in patients with ARNSHL when LOXHD1 is suspected to be the pathogenic gene.

In summary, we identified five novel mutations in *LOXHD1* gene in six members from four Chinese families with congenital nonprogressive sensorineural hearing loss. Both the targeted next generation and Sanger sequencing were used to identify and verify the pathogenic mutations. Genetic analysis revealed that the five novel mutations in *LOXHD1* were involved in the underlying pathogenic mechanism of hearing loss in those families studied, and these results have expanded the spectrum of *LOXHD1* mutations. Future studies are needed to determine the relationship between phenotype and genotype of LOXHD1 gene mutations.

## Figures and Tables

**Table 1:** PCR primer sequences used in the experiments.

Primer	Forward sequence	Reverse sequence
c.2329C>T	5′-GACTGGAGACCTGGGTTGTGT-3′	5′-CATGGGAAACAATGGGTGGTCC-3′
c.5888delG	5′-TCGCTGTAGCCCCAGAATCC-3′	5′-ATGGGCCTCCCCTTCCTACTT-3′
c.611-2A>T	5′-CCAATTCAGGACAAGCAACTGGC-3′	5′-AGAAGAGTGGATGCAGATGGACC-3′
c.277G>A	5′-GGAGGAAGAAGCGGAATGCCA-3′	5′-TCCAGTGGGGAAGTTTAGGGC-3′
c.1255+3A>G	5′-GTTCCTGTTCCTATGCGGGC-3′	5′-ATCTCAGGACTTCTTCCCCTGC-3′

**Table 2:** Summary of clinical data for members in hearing loss families.

Subject	Gender	Age at test (years)	Age at onset	Use of aminoglycoside	PTA (dB) right ear	PTA (dB) left ear	Level of hearing impairment
F098^*∗*^ I-1	Male	38	—	No	16	12.5	Normal
F098^*∗*^ I-2	Female	39	—	No	14	13	Normal
F098^*∗*^II-1	Male	14	Congenital	No	81	84	Profound
F098^*∗*^ II-2	Male	4	Congenital	No	76	88	Severe
F564^*∗*^ II-1	Female	8	Congenital	No	73	70	Severe
F564^*∗*^ II-2	Female	3	Congenital	No	109	100	Profound
SD1226^*∗*^ II-1	Male	8	Childhood	No	95	91	Profound
SD1226^*∗*^ II-2	Male	6	—	No	12.5	12.5	Normal
SD1391^*∗*^ II-1	Female	7	Congenital	No	78	77	Severe

**Table 3:** LOXHD1 gene mutations found in patients with DFNB77.

Mutations	Ethnicity	Age of HL diagnosis	Severity of HL	Progression of HL	Reference
c.71delT (p.L24Rfs^*∗*^74)	Turkish	Congenital or prelingual	Severe or profound	NA	[16]
c.246-1G>C	Japanese	Congenital	Profound	Progressive	[17]
c.277G>A (p.D93N)	Chinese	Congenital	Severe-profound	Stable	This study
c.442A>T (p.K148^*∗*^)	NA	NA	NA	NA	[18]
c.486_487delCTinsGG	Saudi Arabian	NA	NA	NA	[19]
c.611-2A>T	Chinese	3 years	Severe-profound	Stable	This study
c.894T>G (p.Y298^*∗*^)	NA	Congenital	Mild-moderate	NA	[20]
c.1255+3A>G	Chinese	Congenital	Severe-profound	Stable	This study
c.1270+4A>C	Japanese	36 years	Mild	Progressive	[17]
c.1588G>T (p.E530^*∗*^)	Qatari	Childhood	Severe-profound	Progressive	[19]
c.1603C>T (p.R535^*∗*^)	American	Childhood	Mild-moderate	NA	[21]
c.1618dupA (p.T540Nfs^*∗*^24)	Dutch	Congenital—1 year	Moderate-severe	Stable-progressive	[10]
c.1730T>G (p.L577R)	Dutch	Congenital—1 year	Moderate-severe	Stable-progressive	[10]
c.1730T>G (p.L577R)	NA	Congenital	Severe-profound	NA	[20]
c.1751C>T (p.T584M)	Chinese	NA	NA	NA	[6]
c.1828G>T (p.E610^*∗*^)	Dutch	2–4 years	Mild	Stable	[10]
c.1843C>T (p.R615W)	Chinese	NA	NA	NA	[8]
c.1904T>C (p.L635P)	Dutch	2-3 years	Mild	Stable-progressive	[10]
c.1938G>A (p.K646K)	NA	Childhood	Mild-moderate	NA	[20]
c.1938G>A (p.K646K)	American	Childhood	Mild-moderate	NA	[21]
c.2008C>T (p.R670^*∗*^)	Iranian	7-8 years	Mild-profound	Progressive	[20]
c.2329C>T (p.Q777^*∗*^)	Chinese	Congenital	Severe-profound	Stable	This study
c.2641G>A (p.G881R)	Dutch	2–4 years	Mild	Stable	[10]
c.2696G>C (p.R899P)	NA	NA	NA	NA	[20]
c.2696G>C (p.R899P)	Dutch	5 years	Moderate	Stable	[10]
c.2696 G>C (p.R899P)	Dutch	Congenital	Mild	Too young to determine	[10]
c.2726C>T (p.T909M)	Japanese	30 years	Profound	Progressive	[17]
c.2825_2827delAGA (p.K942del)	NA	Childhood	Mild-moderate	NA	[20]
c.2863G>T (p.E955^*∗*^)	Turkish	NA	NA	NA	[22]
c.3061C>T (p.R1021^*∗*^)	Indian	Congenital	Severe	Stable	[10]
c.3061+1G>A	Dutch	Congenital	Moderate	NA	[10]
c.3076G>T (p.V1026F)	Japanese	3 years	Profound	Stable	[23]
c.3169C>T (p.R1057^*∗*^)	Dutch	Congenital	Severe	Stable	[10]
c.3281A>G (p.D1094G)	Chinese	NA	NA	NA	[8]
c.3371G>A (p.R1124H)	Cameroonian	Prelingual	Profound	NA	[20]
c.3571A>G (p.T1191A)	Spanish	Congenital	Severe-profound	NA	[24]
c.3578C>T (p.A1193V)	Japanese	Congenital	Moderate	NA	[17]
c.3596T>C (p.L1199P)	NA	NA	NA	NA	[20]
c.3748+1G>C	Dutch	Congenital	Moderate-severe	Stable -progressive	[10]
c.3834G>C (p.W1278C)	Dutch	5 years	Moderate	Stable	[10]
c.3857G>T (p.G1286V)	Japanese	Congenital	Mild	Progressive	[17]
c.3979T>A (p.F1327I)	Cameroonian	Prelingual	Profound	NA	[20]
c.4099G>T (p.E1367^*∗*^)	NA	Congenital	Severe-profound	NA	[20]
c.4212+1G>A	Japanese	Congenital	Profound	Stable	[25]
c.4212+1G>A	Japanese	Congenital—7 years	Mild-profound	Progressive	[26]
c.4213-1G>A	Japanese	5 years	Mild	NA	[17]
c.4217C>T (p.A1406V)	NA	NA	NA	NA	[18]
c.4217C>T (p.A1406V)	NA	Childhood	Mild-moderate	NA	[20]
c.4375+1G>T	Japanese	3 years	Profound	Stable	[23]
c.4480C>T (R1494^*∗*^)	Turkish	NA	NA	NA	[22]
c.4480C>T (p.R1494^*∗*^)	NA	Congenital	Mild-moderate	NA	[20]
c.4480C>T (p.R1494^*∗*^)	Caucasian	40 years	Severe-profound	Progressive	[27]
c.4480C>T (p.R1494^*∗*^)	Japanese	1–6 years	Moderate -severe	Stable	[25]
c.4480C>T (p.R1494^*∗*^)	NA	Childhood	Severe-profound	NA	[20]
c.4526G>A (p.G1509E)	Caucasian	40 years	Severe-profound	Progressive	[27]
c.4623C>G (p.Y1541^*∗*^)	Czech	Congenital	Severe	NA	[26]
c.4678T>C (p.C1560R)	Dutch	2-3 years	Mild	Stable-progressive	[10]
c.4714C>T (p.R1572^*∗*^)	Ashkenazi Jewish	Congenital-prelingual	Severe-profound	NA	[28]
c.4734C>G (p.Y1578^*∗*^)	Japanese	Congenital	Profound	Progressive	[17]
c.4936C>T (p.R1646^*∗*^)	NA	Childhood	Mild-moderate	NA	[20]
c.5086-3C>A	Japanese	30 years	Severe	Progressive	[17]
c.5545G>A (p.G1849R)	Czech	Congenital	Severe	NA	[26]
c.5608C>T (p.R1870W)	Japanese	36 years	Mild	Progressive	[17]
c.5674G>T (p.V1892F)	Japanese	Congenital—7 years	Mild-profound	Progressive	[29]
c.5734G>A (p.D1912N)	Japanese	30 years	Severe	Progressive	[17]
c.5815G>A (p.D1939N)	Chinese	NA	NA	NA	[6]
c.5869G>T (p.E1957^*∗*^)	Japanese	1–6 years	Moderate-severe	Stable	[25]
c.5885C>T (p.T1962M)	Indian	Congenital	Severe	Stable	[10]
c.5888delG (p.G1963Afs^*∗*^136)	Chinese	Congenital	Severe-profound	Stable	This study
c.5894dupG (p.G1965fs^*∗*^)	Arab	Prelingual	Profound	NA	[30]
c.5933G>A (p.G1978D)	Japanese	32 years	Profound	Progressive	[17]
c.5934C>T (p.G1978 G)	Dutch	Congenital	Mild	Too young to determine	[10]
c.5944C>T (p.R1982^*∗*^)	NA	Congenital	Severe-profound	NA	[20]
c.5948C>T (p.S1983F)	Chinese	Congenital	Profound	Stable	[7]
c.6037G>A (p.G2013R)	Japanese	5 years	Profound	Progressive	[17]
c.6162_6164delCCT (p.F2055del)	NA	Congenital	Severe-profound	NA	[20]
c.6168delC (p.C2057Vfs^*∗*^42)	Japanese	3 years	Severe	Progressive	[17]
c.6353G>A (p.G2118E)	NA	Congenital	Mild-moderate	NA	[20]
c.6353G>A (p.G2118E)	Dutch	Congenital	Moderate	NA	[10]
c.6353G>A (p.G2118E)	Dutch	Congenital	Severe	Stable	[10]
c.6353G>A (p.G2118E)	Dutch	Congenital	Moderate-severe	Stable-progressive	[10]
c.6598delG (p.D2200Mfs^*∗*^22)	NA	Childhood	Severe-profound	NA	[20]

^*∗*^LOXHD1 sequence (RefSeq NM_144612.6) was used as a reference.

## Data Availability

The data used to support the findings of this study are available from the corresponding author upon request.

## Supplementary Materials

Supplementary Materials Supplementary Table 1: summary of the 127 targeted deafness genes. Supplementary Figure 1: the anterior segment eye photographs of family members. The probands in F098^*∗*^ family and their blood relatives showed no abnormalities in the cornea through an ophthalmology examination.
