# Corrigendum to “Five Novel Mutations in LOXHD1 Gene Were Identified to Cause Autosomal Recessive Nonsyndromic Hearing Loss in Four Chinese Families”

**DOI:** 10.1155/2020/9519415

**Published:** 2020-09-28

**Authors:** Xiaohui Bai, Chi Zhang, Fengguo Zhang, Yun Xiao, Yu Jin, Haibo Wang, Lei Xu

**Affiliations:** ^1^Otologic Center, Shandong Provincial ENT Hospital Affiliated to Shandong University, Jinan, China; ^2^Department of Clinical Laboratory, Shandong Provincial Hospital Affiliated to Shandong University, Jinan, China

In the article titled “Five Novel Mutations in LOXHD1 Gene Were Identified to Cause Autosomal Recessive Nonsyndromic Hearing Loss in Four Chinese Families” [1], there were errors in Section 2.3, Section 3.2, footnote of Table 3, and Figure 4. These errors are shown below:

In Section 2.3, Mutation Confirmation by Sanger Sequencing, “LOXHD1 mRNA (RefSeq NM_144612.6)” should say “LOXHD1 mRNA (RefSeq NM_144612.6 and NM_001308013.1)”.

In Section 3.2, Novel Mutations in LOXHD1 Gene Were Demonstrated to Cause ARNSHL, “PLAT 4 domain” should say “the region between PLAT 11 and PLAT 12 domain”.

In the footnote of Table 3, “LOXHD1 sequence (RefSeq NM_144612.6)” should say “LOXHD1 sequence (RefSeq NM_144612.6 and NM_001308013.1)”.

In Figure 4, the location of mutation should be c.1255+3A>G.

The corrected Section 2.3, Section 3.2, footnote of Table 3, and Figure 4 are shown below. Figure 4 is listed as Figure 1 and Table 3 is listed as Table 1.

## 2.3. Mutation Confirmation by Sanger Sequencing

We performed Sanger sequencing to verify the mutations in subjects and 200 controls. PCR was employed to amplify the regions corresponding to these mutations (Table 3). LOXHD1 mRNAs (RefSeq NM_144612.6 and NM_001308013.1) were used as a reference to align the sequences with the Lasergene-SeqMan software.

## 3.2. Novel Mutations in LOXHD1 Gene Were Demonstrated to Cause ARNSHL

Five novel mutations (family F098∗, F564∗, SD1226∗, and SD1391∗) in LOXHD1 gene were identified pathogenic variants based on predictive analysis using PolyPhen2, SIFT, and Mutation Taster. Sanger sequencing was used in all the subjects to verify variants in LOXHD1. The c.2329C>T (p.Q777X) and c.5888delG (p.G1963Afs∗136) mutations were both found in family F098∗ and family F564∗. The c.611-2A>T mutation was verified in family SD1226∗, while c.277G>A (p.D93N) and c.1255+3A>G were verified in family SD1391∗. Sequencing results are shown in Figures 2 and 3, and the schematic diagrams of protein structure are shown in Figure 4.

In general, according to ACMG guidelines [13], the mutations of c.611-2A>T, c.1255+3A>G, c.2329C>T (p.Q777∗), and c.5888delG (p.G1963Afs∗136) were classified as pathogenic; in addition, the mutation of c.277G>A (p.D93N) was classified as likely pathogenic. Moreover, all the mutations were newly identified and never reported previously (Figure 4). Those mutations were absent in all of 200 control subjects with the method of Sanger sequencing.

The mutation of c.2329C>T (p.Q777X) is a nonsense mutation, which leads to a stop codon in PLAT 6 domain. The variant c.277G > A (p.D93N) is also a missense mutation found in PLAT 1 domain. But the mutation of c.5888delG (p.G1963Afs∗136) is a frameshift mutation in PLAT 14 domain, resulting in a truncated protein of LOXHD1. In addition, the mutations of c.611-2A>T and c.1255+3A>G can cause defects in alternative gene splicing of PLAT 2 and the region between PLAT 11 and PLAT 12 domain, respectively. Figure 4 shows all the previously reported mutations in LOXHD1 that cause DFNB77-type deafness, as well as novel mutations identified in this study. These results showed that LOXHD1 mutations are found throughout all PLAT domains.

## Figures and Tables

**Figure 1:**
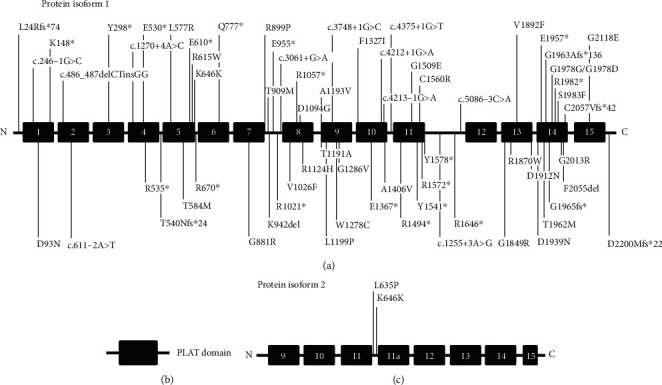
All identified pathogenic variants in LOXHD1 gene associated with DFNB77. (a) Isoform 1 represents LOXHD1 protein NP_653213.6. (b) Schematic representation of PLAT protein domain. (c) Isoform 2 represents LOXHD1 protein NP_001138944.1. Two variants (L635P and splice site variants K646K) only affect the shorter isoform 2. The blue label represents the previously reported mutations causing DFNB77 deafness, while the red label represents novel mutations in this work [14].

**Table 3:** LOXHD1 gene mutations found in patients with DFNB77.

Mutations	Ethnicity	Age of HL diagnosis	Severity of HL	Progression of HL	Reference
c.71delT (p.L24Rfs74)	Turkish	Congenital or prelingual	Severe or profound	NA	[16]
c.246-1G>C	Japanese	Congenital	Profound	Progressive	[17]
c.277G>A (p.D93N)	Chinese	Congenital	Severe-profound	Stable	This study
c.442A>T (p.K148)	NA	NA	NA	NA	[18]
c.486_487delCTinsGG	Saudi Arabian	NA	NA	NA	[19]
c.611-2A>T	Chinese	3 years	Severe-profound	Stable	This study
c.894T>G (p.Y298)	NA	Congenital	Mild-moderate	NA	[20]
c.1255+3A>G	Chinese	Congenital	Severe-profound	Stable	This study
c.1270+4A>C	Japanese	36 years	Mild	Progressive	[17].
c.1588G>T (p.E530)	Qatari	Childhood	Severe-profound	Progressive	[19]
c.1603C>T (p.R535)	American	Childhood	Mild-moderate	NA	[21]
c.1618dupA (p.T540Nfs24)	Dutch	Congenital—1 year	Moderate-severe	Stable-progressive	[10]
c.1730T>G (p.L577R)	Dutch	Congenital—1 year	Moderate-severe	Stable-progressive	[10]
c.1730T>G (p.L577R)	NA	Congenital	Severe-profound	NA	[20]
c.1751C>T (p.T584M)	Chinese	NA	NA	NA	[6]
c.1828G>T (p.E610)	Dutch	2–4 years	Mild	Stable	[10]
c.1843C>T (p.R615W)	Chinese	NA	NA	NA	[8]
c.1904T>C (p.L635P)	Dutch	2-3 years	Mild	Stable-progressive	[10]
c.1938G>A (p.K646K)	NA	Childhood	Mild-moderate	NA	[20]
c.1938G>A (p.K646K)	American	Childhood	Mild-moderate	NA	[21].
c.2008C>T (p.R670)	Iranian	7-8 years	Mild-profound	Progressive	[20]
c.2329C>T (p.Q777)	Chinese	Congenital	Severe-profound	Stable	This study
c.2641G>A (p.G881R)	Dutch	2–4 years	Mild	Stable	[10]
c.2696G>C (p.R899P)	NA	NA	NA	NA	[20]
c.2696G>C (p.R899P)	Dutch	5 years	Moderate	Stable	[10]
c.2696 G>C (p.R899P)	Dutch	Congenital	Mild	Too young to determine	[10].
c.2726C>T (p.T909M)	Japanese	30 years	Profound	Progressive	[17]
c.2825_2827delAGA (p.K942del)	NA	Childhood	Mild-moderate	NA	[20]
c.2863G>T (p.E955)	Turkish	NA	NA	NA	[22]
c.3061C>T (p.R1021)	Indian	Congenital	Severe	Stable	[10]
c.3061+1G>A	Dutch	Congenital	Moderate	NA	[10]
c.3076G>T (p.V1026F)	Japanese	3 years	Profound	Stable	[23]
c.3169C>T (p.R1057)	Dutch	Congenital	Severe	Stable	[10].
c.3281A>G (p.D1094G)	Chinese	NA	NA	NA	[8]
c.3371G>A (p.R1124H)	Cameroonian	Prelingual	Profound	NA	[20]
c.3571A>G (p.T1191A)	Spanish	Congenital	Severe-profound	NA	[24]
c.3578C>T (p.A1193V)	Japanese	Congenital	Moderate	NA	[17]
c.3596T>C (p.L1199P)	NA	NA	NA	NA	[20]
c.3748+1G>C	Dutch	Congenital	Moderate-severe	Stable -progressive	[10]
c.3834G>C (p.W1278C)	Dutch	5 years	Moderate	Stable	[10]
c.3857G>T (p.G1286V)	Japanese	Congenital	Mild	Progressive	[17]
c.3979T>A (p.F1327I)	Cameroonian	Prelingual	Profound	NA	[20]
c.4099G>T (p.E1367)	NA	Congenital	Severe-profound	NA	[20]
c.4212+1G>A	Japanese	Congenital	Profound	Stable	[25]
c.4212+1G>A	Japanese	Congenital—7 years	Mild-profound	Progressive	[26]
c.4213-1G>A	Japanese	5 years	Mild	NA	[17].
c.4217C>T (p.A1406V)	NA	NA	NA	NA	[18]
c.4217C>T (p.A1406V)	NA	Childhood	Mild-moderate	NA	[20]
c.4375+1G>T	Japanese	3 years	Profound	Stable	[23].
c.4480C>T (R1494)	Turkish	NA	NA	NA	[22]
c.4480C>T (p.R1494)	NA	Congenital	Mild-moderate	NA	[20]
c.4480C>T (p.R1494)	Caucasian	40 years	Severe-profound	Progressive	[27]
c.4480C>T (p.R1494)	Japanese	1–6 years	Moderate-severe	Stable	[25]
c.4480C>T (p.R1494)	NA	Childhood	Severe-profound	NA	[20]
c.4526G>A (p.G1509E)	Caucasian	40 years	Severe-profound	Progressive	[27]
c.4623C>G (p.Y1541)	Czech	Congenital	Severe	NA	[26]
c.4678T>C (p.C1560R)	Dutch	2-3 years	Mild	Stable-progressive	[10]
c.4714C>T (p.R1572)	Ashkenazi Jewish	Congenital-prelingual	Severe-profound	NA	[28]
c.4734C>G (p.Y1578)	Japanese	Congenital	Profound	Progressive	[17]
c.4936C>T (p.R1646)	NA	Childhood	Mild-moderate	NA	[20]
c.5086-3C>A	Japanese	30 years	Severe	Progressive	[17]
c.5545G>A (p.G1849R)	Czech	Congenital	Severe	NA	[26]
c.5608C>T (p.R1870W)	Japanese	36 years	Mild	Progressive	[17]
c.5674G>T (p.V1892F)	Japanese	Congenital—7 years	Mild-profound	Progressive	[29]
c.5734G>A (p.D1912N)	Japanese	30 years	Severe	Progressive	[17]
c.5815G>A (p.D1939N)	Chinese	NA	NA	NA	[6]
c.5869G>T (p.E1957)	Japanese	1–6 years	Moderate-severe	Stable	[25]
c.5885C>T (p.T1962M)	Indian	Congenital	Severe	Stable	[10]
c.5888delG (p.G1963Afs136)	Chinese	Congenital	Severe-profound	Stable	This study
c.5894dupG (p.G1965fs)	Arab	Prelingual	Profound	NA	[30]
c.5933G>A (p.G1978D)	Japanese	32 years	Profound	Progressive	[17]
c.5934C>T (p.G1978 G)	Dutch	Congenital	Mild	Too young to determine	[10]
c.5944C>T (p.R1982)	NA	Congenital	Severe-profound	NA	[20]
c.5948C>T (p.S1983F)	Chinese	Congenital	Profound	Stable	[7]
c.6037G>A (p.G2013R)	Japanese	5 years	Profound	Progressive	[17]
c.6162_6164delCCT (p.F2055del)	NA	Congenital	Severe-profound	NA	[20]
c.6168delC (p.C2057Vfs42)	Japanese	3 years	Severe	Progressive	[17]
c.6353G>A (p.G2118E)	NA	Congenital	Mild-moderate	NA	[20]
c.6353G>A (p.G2118E)	Dutch	Congenital	Moderate	NA	[10]
c.6353G>A (p.G2118E)	Dutch	Congenital	Severe	Stable	[10]
c.6353G>A (p.G2118E)	Dutch	Congenital	Moderate-severe	Stable-progressive	[10]
c.6598delG (p.D2200Mfs22)	NA	Childhood	Severe-profound	NA	[20]

^∗^LOXHD1 sequence (RefSeq NM_144612.6 and NM_001308013.1) was used as a reference.
