# Supplementary material for: Five Novel Mutations in LOXHD1 Gene Were Identified to Cause Autosomal Recessive Nonsyndromic Hearing Loss in Four Chinese Families
Source: Biomed Res Int. 2020 Feb 18;2020:1685974. doi: 10.1155/2020/1685974 (PMC7049443; doi:10.1155/2020/1685974)

Supplementary Table 1. Summary of the 127 targeted deafness genes.

Gene	NS/S	Inheritance	mRNA	Exons
ACTG1	NS	AD	NM_001199954.1	6
ALMS1	S	AR	NM_015120.4	23
ATP2B2	NS	AR	NM_001001331.2	23
BSND	NS/S	AR	NM_057176.2	4
CACNA1D	NS/S	AR	NM_000720.3	49
CCDC50	NS	AD	NM_178335.2	12
CDH23	NS/S	AR	NM_022124.5,	70
CEACAM16	NS	AD	NM_001039213.3	7
CHD7	S	AD	NM_017780.3	38
CLDN14	NS	AR	NM_144492.2	3
CLRN1	NS/S	AR	NM_174878.2	3
COCH	NS	AD	NM_001135058.1	11
COL11A1	S	AR/AD	NM_001854.3	67
COL2A1	S	AD	NM_033150.2	53
COL4A3	S	AR	NM_000091.4	52
COL4A4	S	AR	NM_000092.4	48
COL4A5	S	X-linked	NM_000495.4	51
COL9A1	S	AR	NM_001851.4	38
COL9A2	S	AR	NM_001852.3	32
CRYM	NS	AD	NM_001888.4	10
DFNA5	NS	AD	NM_004403.2	10
DFNB31	NS/S	AR	NM_015404.3	12
DFNB59/PJVK	NS	AR	NM_001042702.3	7
DIABLO	NS	AD	NM_019887.5	7
DIAPH1	NS	AD	NM_005219.4	28
DIAPH3	NS	AD	NM_001258366.1	27
DLX5	S	AD	NM_005221.5	3
DSPP	NS	AD	NM_014208.3	5
EDN3	S	AD	NM_207032.2	5
EDNRB	S	AD	NM_000115.3	8

ESPN	NS	AR/AD	NM_031475.2	13
ESRRB	NS	AR	NM_004452.3	11
EYA1	S	AD	NM_000503.5	18
EYA4	NS	AD	NM_004100.4	20
FGF3	S	AR	NM_005247.2	3
FGFR1	S	AD	NM_023110.2	18
FGFR2	S	AD	NM_000141.4	18
FGFR3	S	AD	NM_000142.4	18
FOXI1	NS/S	AR/AD	NM_012188.4	2
GATA3	S	AD	NM_001002295.1	6
GIPC3	NS	AR	NM_133261.2	6
GJA1	NS	AR	NM_000165.4	2
GJB2	NS	AR/AD	NM_004004.5	2
GJB3	NS	AR/AD	NM_024009.2	2
GJB6	NS	AR/AD	NM_001110219.2	5
GLI3	S	AD	NM_000168.5	15
GPR98	NS/S	AR	NM_032119.3	90
GPSM2	NS	AR	NM_013296.4	15
GRHL2	NS	AD	NM_024915.3	16
GRXCR1	NS	AR	NM_001080476.2	4
HGF	NS	AR	NM_000601.4	18
HOXA1	S	AR	NM_005522.4	2
HOXA2	S	AR	NM_006735.3	2
IGF1	S	AR	NM_001111283.1	5
ILDR1	NS	AR	NM_001199799.1	8
KCNE1	NS/S	AR	NM_000219.5	4
KCNJ10	NS/S	AR	NM_002241.4	2
KCNQ1	NS/S	AR	NM_000218.2	16
KCNQ4	NS	AD	NM_004700.3	14
LHFPL5	NS	AR	NM_182548.3	4
LOXHD1	NS	AR	NM_144612.6	40
LRP2	S	AR	NM_004525.2	79
LRTOMT	NS	AR	NM_145309.5	6

MARVELD2	NS	AR	NM_001038603.2	7
MIR96	NS	AD	NR_029512.1	
MITF	S	AD	NM_198159.2	10
MSRB3	NS	AR	NM_198080.3	6
MT-RNR1	NS	MT	—	
MT-TE	S	MT	—	
MT-TK	S	MT	—	
MT-TL1	S	MT	—	
MT-TS1	NS	MT	—	
MYH14	NS	AD	NM_001077186.1	42
MYH9	NS	AD	NM_002473.5	41
MYO15A	NS	AR	NM_016239.3	66
MYO1A	NS	AD	NM_001256041.1	29
MYO3A	NS	AR	NM_017433.4	35
MYO6	NS	AR/AD	NM_004999.3	35
MYO7A	NS/S	AR/AD	NM_000260.3	49
NDP	S	X-linked	NM_000266.3	3
OPA1	S	AD	NM_015560.2	29
OTOA	NS	AR	NM_144672.3	28
OTOF	NS	AR	NM_194248.2	47
OTOG	NS	AR	NM_001277269.1	55
PAX2	S	AD	NM_003987.3	11
PAX3	S	AD	NM_181457.3	8
PCDH15	NS/S	AR	NM_001142765.1	32
PDSS1	S	AR	NM_014317.3	12
PDZD7	NS/S	AR	NM_001195263.1	17
PHEX	S	X-linked	NM_000444.5	22
POU3F4	NS	X-linked	NM_000307.4	1
POU4F3	NS	AD	NM_002700.2	2
PRPS1	NS	X-linked	NM_002764.3	7
PRRX1	S	AR	NM_022716.3	4
PTPRQ	NS	AR	NM_001145026.1	45
RDX	NS	AR	NM_001260492.1	16

SEMA3E	S	AR	NM_012431.2	17
SERAC1	S	AR	NM_032861.3	17
SERPINB6	NS	AR	NM_001297699.1	7
SIX1	NS/S	AD	NM_005982.3	2
SIX5	S	AD	NM_175875.4	3
SLC17A8	NS	AD	NM_139319.2	12
SLC19A2	S	AR	NM_006996.2	6
SLC26A4	NS/S	AR	NM_000441.1	21
SLC26A5	NS	AR	NM_198999.2	20
SLC4A11	S	AR	NM_001174090.1	20
SMAD4	S	AD	NM_005359.5	12
SMPX	NS	X-linked	NM_014332.2	5
SNAI2	S	AD	NM_003068.4	3
SOBP	S	AR	NM_018013.3	7
SOX10	S	AD	NM_006941.3	4
SOX9	S	AD	NM_000346.3	3
STRC	NS	AR	NM_153700.2	29
TCOF1	S	AD	NM_001135244.1	26
TECTA	NS	AR/AD	NM_005422.2	23
TIMM8A	NS/S	X-linked	NM_004085.3	2
TJP2	NS	AD	NM_004817.3	23
TMC1	NS	X-linked	NM_138691.2	24
TMIE	NS	AR	NM_147196.2	4
TMPRSS3	NS	AR	NM_024022.2	13
TNFRSF11B	S	AD	NM_002546.3	5
TPRN	NS	AR	NM_001128228.2	4
TRIOBP	NS	AR	NM_138632.2	8
USH1C	NS/S	AR	NM_005709.3	21
USH1G	NS/S	AR	NM_173477.4	3
USH2A	NS/S	AR	NM_206933.2	72
WFS1	NS/S	AR/AD	NM_006005.3	8

NS: Non-Syndromic; S: Syndromic; AR: Autosomal Recessive; AD: Autosomal Dominant; MT: Mitochondrial Inheritance

Supplementary figure 1

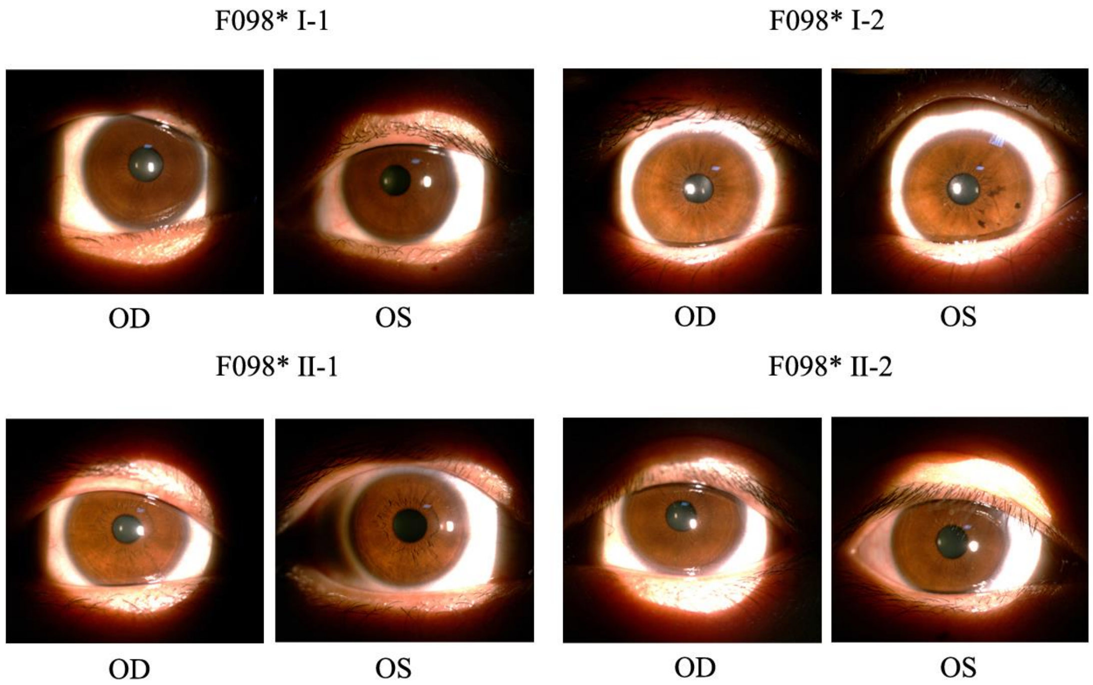

Supplement: Supplementary Materials — Supplementary Table 1: summary of the 127 targeted deafness genes. Supplementary Figure 1: the anterior segment eye photographs of family members. The probands in F098∗ family and their blood relatives showed no abnormalities in the cornea through an ophthalmology examination. [file 1685974.f1.pdf]
